# Conference report: Biocuration 2021 Virtual Conference

**DOI:** 10.1093/database/baac027

**Published:** 2022-04-09

**Authors:** Federica Quaglia, Rama Balakrishnan, Susan M Bello, Nicole Vasilevsky

**Affiliations:** Institute of Biomembranes, Bioenergetics and Molecular Biotechnologies, National Research Council (CNR-IBIOM), Via Giovanni Amendola, 122/O, Bari 70126, Italy; Department of Biomedical Sciences, University of Padova, Via Ugo Bassi, 58/B, Padova 35131, Italy; Genentech, South San Francisco, 1 DNA Way, CA 94080, USA; Jackson Laboratory, 600 Main Street, Bar Harbor, ME 04609, USA; Translational and Integrative Sciences Laboratory, University of Colorado Anschutz Medical Campus, Fitzsimons Building, 1300 E 17th Place, Aurora, CO 80045, USA

## Abstract

The International Society for Biocuration (ISB) aims to promote the field of biocuration and provide a community forum for information exchange and networking. Over the past 14 years, the ISB has hosted annual international conferences, entirely dedicated to the field of biocuration, that rotate between regions across the world. These meetings bring together biocurators from various roles, including database curators, bioinformaticians, ontology developers and students. Due to the ongoing global pandemic, the 14th Annual ISB Biocuration Conference (ISB2021) was held virtually in the form of four sessions and one workshop over the course of the year. Each of the four virtual sessions included panel discussions covering (i) The Future of Biocuration, (ii) Career paths and projections in Biocuration, (iii) Addressing Implicit or Unconscious Bias: Equity, Diversity and Inclusion and (iv) Strategic planning. Here we report on highlights from the virtual conference and share some of the ideas and future goals of the ISB.

Database URL:https://www.biocuration.org/14th-annual-biocuration-conference-virtual/

## Introduction

The field of biocuration is a relatively new, niche field that intersects subdomains of biology and data science, computational biology and bioinformatics. The primary role of professional biocurators is to extract knowledge from biological data and convert it into a structured, computable form via manual, semi-automated and automated methods. The International Society for Biocuration (ISB, https://www.biocuration.org/) is a professional society which was formed in 2009 by a group of biocurators that aimed to promote and support the interests of professional biocurators in the field. The Society is overseen by an elected Executive Committee composed of nine members, and there are formal memberships in the society—currently including 232 members—although anybody in the community is welcome to participate in most of the activities. The goals of the society are to define and promote the work of biocurators, foster connections with user communities to ensure that databases and accompanying tools meet specific user needs, promote communication and exchanges between curators (meetings, workshops and training) and encourage best practices by providing documentation on standards and annotation procedures.

The first ISB conference was held in Asilomar, CA, in 2005 and international conferences have been held annually ever since, rotating throughout the world, alternating between the Americas, Europe and Asia/Australia each year. A list of past conferences is available on our website (the link is available in Table 1 along with other useful links mentioned related to the ISB and mentioned in this article). In 2021, we held our first fully virtual conference due to the ongoing coronavirus disease 2019 pandemic, which was chaired by Rama Balakrishnan and co-chaired by Susan Bello. The virtual format offered a lot of opportunities for increased participation and accessibility for participants from groups or countries who were unable to travel, but also challenges with time zones and lack of face-to-face social interaction.

**Table 1.** Links to the ISB’s website pages and YouTube channel

**ISB’s website links**	
ISB’s website	https://www.biocuration.org/
ISB’s subcommittees	https://www.biocuration.org/about/isb-ec-subcommittees/
ISB2021 Biocuration conference (virtual)	https://www.biocuration.org/14th-annual-biocuration-conference-virtual/
ISB2022 Biocuration conference (virtual)	https://www.biocuration.org/15th-annual-biocuration-conference-virtual/
1st UK-local biocuration conference	https://www.biocuration.org/1st-local-uk-biocuration-conference-may-2022/
List of past ISB conferences	https://www.biocuration.org/community/conferences/international-biocuration-meetings/
Biocuration-related training materials	https://www.biocuration.org/dissemination/biocuration-training-materials/
Biocuration Generic Job Description	https://www.biocuration.org/community/biocuration-generic-job-description/
Job openings in Biocuration	https://www.biocuration.org/community/jobs/
ISB’s YouTube channel links	
ISB YouTube channel	https://www.youtube.com/channel/UCNLZMHYSuWSIjoOinpAxo_Q
Session 1 (13 April 2021) recording	https://www.youtube.com/watch?v=A8NYDNZ_9Fo&t=26s
Session 2 (15 June 2021) recording	https://www.youtube.com/watch?v=wmFf0aq6VNfg&t=497s
Session 3 (17 August 2021) recording	https://www.youtube.com/watch?v=wBf4V-G23No&t=165s
Session 4 (5 October 2021) recording	https://www.youtube.com/watch?v=2Yowgtt4TSc&t=930s

## Meeting highlights

The meeting format for the 14th Annual Biocuration Conference (ISB2021) consisted of four virtual sessions of 2–3 h each, spread throughout the year, and one virtual workshop. The format adopted received an overall positive feedback from the attendees of the conference, which highlighted how short, 2-h sessions several weeks apart were easier to fit into remote/in-presence work and family commitments than usual conference formats. Members of the ISB community were invited to submit abstracts for consideration for talks, and talks were selected for 15-min presentations at the first three sessions. At the final session, we hosted the Annual General Meeting (AGM, described in the following section), invited talks from the Biocuration Award winners and hosted a poster session. Each session included a panel discussion on various topics, which are highlighted below. The session presentations and speakers are outlined in Table 2. All of the sessions were recorded and made freely available online in the dedicated ISB YouTube channel. A booklet collecting the abstracts fromcollecting the abstracts from the invited speakers’ talks and poster session presentations is available in Zenodo (1).

**Table 2.** Fifteen-minute presentations and presenters at each virtual session of the Biocuration 2021 (ISB2021) virtual conference

Presentation title	Presenter
**Session 1**	13 April 2021
A neural network based pipeline to classify publications for UniProtKB protein entries	Patrick Ruch
Addition of RNA Seq Data and New Search Utilities to the Mouse Gene Expression Database (GXD)	Constance Smith
A structured model for immune exposures	Randi Vita
APICURON: a database to credit and acknowledge the work of biocurators	Federica Quaglia
**Session 2**	15 June 2021
Automatic Consistency Assurance for Literature-based Gene Ontology Annotation	Jiyu Chen
Biocuration training for undergraduate students: challenges and opportunities	Sushma Naithani
A Resource for the Network Representation of Cell Perturbations Caused by SARS-CoV-2 Infection	Livia Perfetto
Functional annotation of specific protein products in UniProtKB/Swiss-Prot	Sylvain Poux
**Session 3**	17 August 2021
Identifying New Chemical and Genetic Terms for Inclusion in the MeSH Vocabulary	Nicholas Miliaras
neXtProt function prediction community pages	Paula Duek
wwPDB Biocuration: On the Front Line of Structural Biology	Jasmin Young
DrugMechDB: a database of drug mechanisms, Integrated Structural and Computational Biology Scripps Research	Anna Tanska
**Session 4**	5 October 2021
AGM for the ISB	Nicole Vasilevsky
Biocuration Award talk: Exceptional Contributions to Biocuration	Amos Bairoch
Biocuration Award talk: Biocuration Career Award	Anne Niknejad
Poster session (Abstract booklet, (1))	

### Annual General Meeting

The ISB hosts an AGM each year to provide an overview of the work by the Executive Committee from the past year. The ISB is known for fostering and building connections among the members, initially thanks to the meetings that took place over the years, and now including additional venues that facilitate our interactions, such as a mailing list, newsletter, social media presence, a Slack workspace and a dedicated Outreach, Training and Communication subcommittee. The ISB gives out awards, microgrants and fellowships for travel or exchanges, which have played a crucial role in raising awareness on the centrality of biocuration careers inside the scientific community and in supporting knowledge exchange between biocurators from different groups.

Over the years, the ISB has been growing to be more inclusive and diverse and focused on developing and implementing a code of professional conduct. The introduction of the Equity, Diversity and Inclusion (EDI) subcommittee, composed not only of Executive Committee (EC) members but of the greater ISB members too, increased the ability of community members to volunteer in the activities of the ISB. The society is now also exploring new ways to promote a variety of professional experiences by engaging biocurators in poorly represented geographical areas and by welcoming graduate students, by considering introduction of a dedicated ‘students section’.

**Figure 1.**
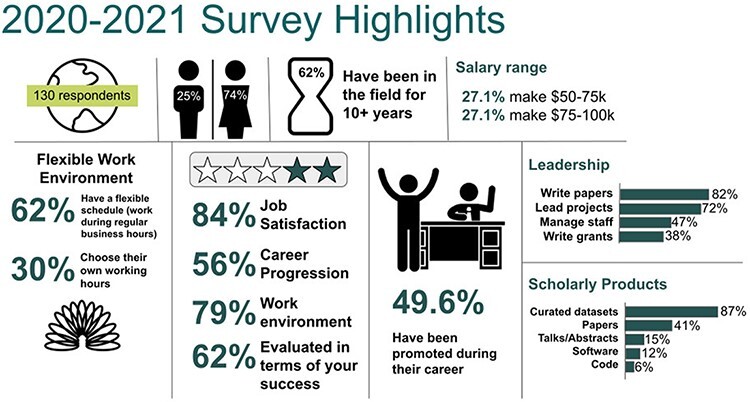
Results of the survey assessing the work of biocurators; the survey was presented during the 2021 AGM of the ISB.

In an effort to assess the work of biocurators, the ISB sent out a survey during the last year that shed light on biocuration-related work positions, satisfaction, work environment, leadership levels and scholarly products. The results of the survey (Figure 1), presented during the 2021 AGM, are available in Zenodo (2) and summarized below.

The survey had over 130 respondents—with a gender balance of 74% female and 25% male. Interestingly, the majority of respondents have been in the field for over 10 years (62%), suggesting their satisfaction and identifying biocuration as a stable career choice. For what concerns the salary range of biocurators, half of respondents (54.2%) earn between 50 and 100k a year in US dollars. Further inquiring on the work environment highlighted some flexibility in the work schedule (identified as flexibility during regular business hours) for 62% of biocurators; an additional 30% are actually able to choose their own working hours. Among the biocurators who participated in the survey, 84% are satisfied with their job, with their work environment (79%), professional success (62%) and career progression (56%)—people are overall highly satisfied. Less than half of respondents (49.6%) said they have been promoted during their career. In terms of leadership opportunities, we were able to identify four main areas of leadership for biocurators, namely manuscript drafting and publication (82%), project leading (72%), staff management (47%) and writing grant applications (38%), pointing to the involvement of biocurators in managerial positions and further supporting the high rate of satisfaction in biocuration careers. Finally, the survey identified the five main types of scholarly products generated by biocurators: curated datasets (87%), publications (41%), talks at conferences (15%), software (12%) and code snippets (6%). The relatively low proportion of respondents who had generated publications indicates a possible need to increase the number of articles published by biocurators.

### Poster session

Poster sessions at conferences are often a favorite among attendees, as they give researchers an opportunity to present their work in a more informal format, allow for impromptu conversations and new connections to form and usually provide some form of refreshments. Finding a suitable substitute for face-to-face poster sessions was a challenge, as virtual formats cannot recapitulate the in-person experience. We used the new web-conferencing software Gather (https://www.gather.town/) to host our poster session, which provided a video-game-like and interactive environment to display the posters and enable informal social interactions.

## Panel session highlights

At each virtual session, we hosted panel discussions with invited speakers that covered various topics of interest to the community.

### Session 1: the future of biocuration

At the inaugural conference session, a group of panelists discussed **‘**the future of biocuration**’**. The panel was moderated by Rama Balakrishnan, who has served on the ISB Executive Committee since 2017, and was the co-chair (along with Susan Bello from the Jackson Laboratory) of the Biocuration 2021 conference. Rama was joined by four panelists from various roles in academia and industry to discuss what is in store for our community (Table 3).

**Table 3.** Panelists of the first session of ISB2021 virtual conference; the link to the session recording is available in Table 1

Panelist	Affiliation
Rama Balakrishnan, Moderator	Genentech, California, USA
Carol Bult	The Jackson Laboratory, Maine, USA
James Malone	SciBite, Cambridge, UK
Kambiz Karimi	Myriad Women’s Health, California, USA
Sandra Orchard	EBI, Cambridge, UK

#### What is curation: distilling knowledge from information

Rama initiated the discussion with the fundamental and relevant question, ‘what does the word curation mean to you?’. Working in the biocuration field, many curators can probably relate to this question, which is frequently asked by people who are outside this field. The role of a curator at a museum, for example, may be more familiar, but biocuration is a less well-understood field. Rama, who has held varying roles as a curator (academic and industry), tried to delineate how the actual task of curation may differ among us. Sandra Orchard, from European Bioinformatics Institute (EBI), shared a classical definition of ‘turning unstructured data into structured searchable data’, but recognized this is not always true as, while some curation tasks involve making data more structured, text-minable and machine-readable, the outcome of data curation does not always result in completely structured data. Carol Bult from Mouse Genome Informatics (MGI) defined curation as ‘applying semantic standards to ensure data findability and aggregation’.

Coming from the industry perspective, both Kambiz Karimi (Myriad Women’s Health) and James Malone (SciBite) agreed. Curation involves meaning-based capture and structuring of content using controlled vocabularies. Data curation can also include data cleaning, which is often a pre-curation task. Curation can help improve and enrich data interpretability and ultimately add value. It allows for enhanced search, querying, semantic integration and meta-analysis.

#### How can we ensure quality?

Given that the panelists all agreed on a high-level definition of curation, Rama then asked about ensuring data quality. ‘What does good quality mean and what are metrics to assess quality?’ Different quality control (QC) and quality assurance processes apply, depending on the type of curation that is being done, whether you are curating tax forms or curating the mouse biology literature. Some processes that were discussed by Carol and others included intercurator checks, crowdsourcing feedback from downstream users, practices to ensure collaboration, regression testing to ensure continuity and consistency across datasets. Sandra pointed out that curators cannot be all things to everything and stressed the importance of specialist databases with curators who are domain experts who can take the first pass at the curation and build re-processing pipelines or scoring mechanisms to export high-quality subsets to other data resources.

James and Rama noted how detecting outliers can assist with quality checks. However, it may not always be easy to detect the outliers without the expert knowledge in a specific area. For example, Rama curates patient data at Genentech and once came across data reporting a patient had a 100°C fever (rather than 100°F), which was easy to spot as an error. However, in a more complicated clinical use case, detecting erroneous data points may not be so obvious and require more specialized knowledge.

Kambiz shared that Myriad has several QC approaches, including a peer review process, a dedicated program where curators spot check each other’s work and a quality check process that compares their classification to previous classifications from the community.

Sandra noted the importance of researchers collaborating with curators prior to publication. She shared an anecdote where an author published a paper with an erroneous dataset, a simple mistake where a row in a spreadsheet had been accidentally deleted, causing nonsensical results. The curator picked this up and contacted the author, who was able to correct it. This speaks to the importance of pre-submitting data to the database before publication and the important role a curator can play in the research community.

#### Opportunities with machine learning and automation

While a lot of biocuration is done manually, more and more processes and workflow are being automated, with text mining, machine learning (ML), natural language processing (NLP) and artificial intelligence (AI). The panel was asked their opinion on ‘how AI and ML will affect the work of biocurators?’. Sandra assured us that ML will enhance our work but is not concerned that it will replace human curation. Data are too messy, the literature is too unstructured and human review and curation is going to be needed in the foreseeable future. James echoed her sentiments in saying that ‘[Machine Learning] will become an assistant, it will not replace subject matter experts who are biologists, scientists, curators. It will play a role in helping us’. James sees it as an opportunity for biocuration, where we should work to exploit advances in deep learning, noting the importance of biocuration is more pronounced now than ever. We can train AI to aid in biocuration and we can work together. In addition, quality ML/AI requires training sets that have been human-curated, and the advances of these technologies will require more curators; this is a new opportunity for this community. Carol agreed, but brought up the point that there may be the perception that these technologies are advanced to the point where curators can be replaced. This is causing challenges with funding for biocuration due to the notion that ML can do all or most of what human curators do. While ML can assist with making biocuration scalable, we need to do better as a community at communicating how these things interrelate and feed off each other.

Biocuration has never been more valuable than it is now and yet under-appreciated. It’s something the Society can help us tackle, this perception, and [can help us] articulate how manual and machine learning biocuration can go hand and hand.**—**Carol Bult

#### Approaching authors

An audience member inquired whether database curators approached authors for clarification about their published data and whether authors were responsive. Kambiz shared that they did approach authors when there was ambiguity with the content or data in an article. Sandra concurred and alluded to the challenge with time dependencies; if a paper was recently published (1 year to 18 months ago), they frequently got a response. If a paper is over 3 years old, in general, they are less likely to get a reply, as the first author may have moved on and the Principal Investigator (PI) is unfamiliar with the details of the data.

This may speak to an opportunity to better train researchers in becoming familiar with curation methods and standards, to allow for unambiguous reporting in their publications. Requirements to share data at the time of publication will also help address this need.

#### Getting the journals involved

This led to the next question about working with the journals to publish data in a more structured way. Carol has had some experience working with journals in the mouse community, who are careful about publishing mouse names with the accepted terminology and nomenclature. She did mention that sometimes there is push back as to whether the recommended standard is the accepted standard, and whether this is going to evolve or change in the future.

This is an opportunity for a systematic community approach; the ISB should promote standards adoption to the journals.

Sandra pointed out that a challenge with approaching journals to use our standards is the sheer number of journals. A more targeted approach may be more appropriate. For example, the proteomics community was successful in getting a restricted number of journals in their field to require data sharing to ProteomeXchange (http://www.proteomexchange.org/) prior to publication.

Sandra also recommended that we first talk among ourselves as a community and define our needs and what standards to adopt and promote and then approach the journals.

#### The elephant in the room: funding

In recent years, National Institutes of Health (NIH) funding has decreased to various databases. How do we sustain our own careers and train the next generation of curators?

Kambiz felt it is easier to justify the need for curation due to the regulatory aspect of his industry. Even if there are NLP-based processes to extract gene to disease relationships, manual review will always be needed. He foresees that automated processes will assist with manual curation going forward.

Carol emphasized that we need to promote how important curation is to data science. Data science is recognized as an important field; therefore, we should frame curation within its role in data science. We have to be better about explaining return on investment in curation—‘what can we do when data is curated, and we wouldn’t be able to do, if it wasn’t?’ She pointed out that the reality that biocuration is considered infrastructure, which is largely ignored, until it is broken. As a Society, can we demonstrate the impact that biocuration has on advancing data science?

Sandra concluded that we need to make ourselves more visible and we need people outside the community to understand the relevance of what we do. The biocuration community needs to work together to not duplicate efforts and to align to standars. This should be done by using specialist databases for initial analysis and data cleaning, using the baseline resources like accession numbers and showing good examples of careful curation.

### Session 2: career paths and projections in biocuration

Peter Uetz, Ph.D. from the Virginia Commonwealth University chaired the panel discussion which focused on career paths and projections in biocuration and hosted three panelists: Pankaj Jaiswal, Ph.D., Professor in Plant Genomics at Oregon State University (OSU) in Corvallis, Oregon; Tanya Berardini, Ph.D., co-founder and Chief Scientific Officer at Phoenix Bioinformatics in Newark, California; and Nicola Mulder, Ph.D., Professor of Computational Biology at the University of Cape Town in South Africa (Table 4).

**Table 4.** Panelists of the second session of ISB2021 virtual conference; the link to the session recording is available in Table 1

Panelist	Affiliation
Peter Uetz, Moderator	Virginia Commonwealth University, VA, USA
Pankaj Jaiswal	OSU, Oregon, USA
Tanya Berardini	Phoenix Bioinformatics, California, USA
Nicola Mulder	University of Cape Town, South Africa

#### Panelist paths in Biocuration

Dr Tanya Berardini entered the biocuration field after completing a Ph.D. and a postdoc when she joined The Arabidopsis Information Resource (TAIR) as a curator. When TAIR underwent a funding crisis after many years of serving the plant genome community, Dr Berardini and her colleagues founded the nonprofit Phoenix Bioinformatics which developed a sustainable model to support the TAIR database through subscriptions and has subsequently expanded into assisting other databases and resources to address funding issues, through subscription and membership models. Dr Beradini’s career path is unique, as she initially performed database curation for a single resource, TAIR, and now also works in an entrepreneurial position. She has learned various aspects about running a business [such as human resources (HR), insurance requirements and contract negotiation] as well as curation in additional domains outside of plant biology. Dr Beradini noted that her detail-oriented curation skills and experience with databases were very transferable to the business world.

Dr Pankaj Jaiswal’s work on sequencing plant molecules (his initial training was in biochemistry and plant molecular biology) prompted his interest in bioinformatics analyses and genome biology curation. He currently runs a wet lab (‘on the bench’) and a dry lab (‘at the computer’) at OSU in the Comparative Plant Genomics department. Dr Jaiswal leads the curation efforts for the Gramene database and the Planteome projects, which require the creation of ontologies for the standardization of plant characteristics such as gene function, phenotypes, pathways and gene expression. Dr Jaiswal started curating during his basic science training as he read papers; he learned about specific subjects and synthesized information to address biological questions. His efforts to facilitate the synthesis of information and ease of interpretation, search and access included networking with peers, including Gene Ontology and Model Organism Database curators, and brought him to the field of biocuration. Dr Jaiswal currently trains his students, postdocs and researchers to apply data standards and learn the curation process to build upon the foundations laid by the biocuration community.

Dr Nicola Mulder holds a Ph.D. from the University of Cape Town in South Africa, where she did basic science research and studied molecular biology of infectious diseases, which ultimately led her to bioinformatics. She became a curator at EBI, first at SwissProt and then as part of the InterPro project, which she went on to lead. Dr Mulder currently leads the Pan African Bioinformatics Network for the Human Heredity and Health in Africa (H3Africa) in Cape Town, which supports bioinformatics and genomic analysis in Africa. Her team brought together a global community of experts, including clinicians, biocurators and ontologists, which led to the development of the Sickle Cell Disease Ontology in response to the need to standardize information around sickle cell disease and the Hearing Impairment Ontology. Dr Mulder and her team’s curation efforts include standardizing phenotype data for research cohorts and curating genomic data for African relevance, such as curating single nucleotide polymorphisms from African populations and curating diseases that are relevant to Africans.

#### Becoming a biocurator

The field of biocuration is still relatively new and small; colleges and universities do not typically offer a degree in biocuration. Therefore, the path to becoming a biocurator rarely follows a straightforward trajectory like many other fields, as many biocurators are subject matter experts in various subdomains of biology who completed a Ph.D. in a biological area or have a background in some aspect of computer science or semantic technologies and have an interest in standardizing data. Our panelists shared some suggestions for those interested in joining the field:

- **Draw on your area of expertise**: Most databases focus on specific subject areas and expert community contributions (such as contribution to open biomedical ontologies and all of the OBO Foundry ontologies) are always needed, welcomed and greatly appreciated. If you notice missing information or content in a database, reach out and share your knowledge.
- **As a researcher, curate your data before it is published:** Work with the databases to make sure your data are prepared in a proper format for completeness and efficiency before you publish. Dr Berardini mentioned that over 10 000 labs work on Arabidopsis, creating a massive backlog of papers to curate. Structuring data before and at the time of publication dramatically assists with the curation process.
- **Volunteer at databases**: If you have expertise in a particular field, contact the databases directly to discuss opportunities to contribute. Volunteering can be beneficial to build your experience, provide contributions to biocuration efforts and provide networking opportunities within the community. In addition, volunteering can reveal whether the field is right for you. Biocuration requires a particular personality, including attention to detail and a desire to organize. While some people derive extreme satisfaction from it, others can find it quite tedious. Dr Berardini noted, ‘if through volunteering, you find biocuration brings you joy, this is the right career for you’. A dedicated page to help researchers looking for volunteering opportunities in biocuration is available on the ISB website.
- **Participate in hackathons, data jamborees and biomedical competitions:** These events bring together researchers across various career stages, from junior biologists to practicing clinicians, and are opportunities to network, build your curriculum vitae and contribute to impactful work. Examples are biomedical competitions like Dream Challenges, hackathons, data jamborees, face-to-face meetings and online events hosted by Dr Mulder to facilitate community curation of H3Africa projects.
- **Do as much training as you can**: Courses are available, such as massively open online courses and college courses. A dedicated page for training materials useful to biocurators—ranging from biocuration, databases, ontologies to data management, programming and bibliometrics—is available on the ISB website. New training opportunities will be explored and promoted by the ISB in the coming years.
- **Build your skill set:** Search for job advertisements to determine what qualifications are needed, and work toward enhancing your skill set and competencies that meet job requirements. As an outcome of the Careers in Biocuration Workshop at the Biocuration 2018 conference, we created a generic position description for a biocuration profession on the ISB website.

#### Biocuration career opportunities

A lot of opportunities exist in the biocuration field: biocuration in academia, which may entail biocuration for grant-funded database projects and ontology development, such as the work of Dr Jaiswal; community-based bioinformatics and curation projects, such as those led by Dr Mulder; and biocuration in a nonprofit business setting, as Dr Berardini’s work at Phoenix Bioinformatics. Biocuration opportunities are also available in the industry as companies are recognizing the importance of curating and standardizing data (for example, standardizing clinical trial data), in government agencies and even as independent consultants.

The skills gained as biocurators, such as attention to detail, the ability to take in and synthesize data, and computational skills, are very valuable and can be translated to different areas, such as other areas of science or technologies.

Biocuration is a growing field and we anticipate that as the amount of biological data being generated increases, so will the demand for curators. The ISB aims to promote the field and support our community through offering dissemination of job openings (see regular posts on our website), training opportunities and networking. The ISB also promotes collaborations and exchanges between biocuration groups and offers funding for exchange fellowships. This fellowship will fund members to visit another laboratory or organization for training or knowledge sharing.

Researchers have the opportunity to better structure their datasets, share their data in repositories and better structure the content that they publish; however, they are often unaware of the career opportunities in biocuration. We have not only an opportunity to promote the biocuration field, but also the responsibility to train the future generations, provide knowledge transfer and have succession plans for those coming up after us.

### Session 3: addressing implicit or unconscious bias

Sushma Naithani, Associate Professor Senior Research and Lead Biocurator for Plant Reactome at OSU led a Workshop on Addressing Implicit or Unconscious Bias organized by the EDI committee of the ISB. Three invited panelists joined the discussion: Laurie Goodman, Publishing Director, GigaScience Press; Yasmin Alam-Faruque, Senior Biocurator at Healx; and Varsha Khodiyar, Data Curation Manager at Springer Nature (Table 5).

**Figure 2.**
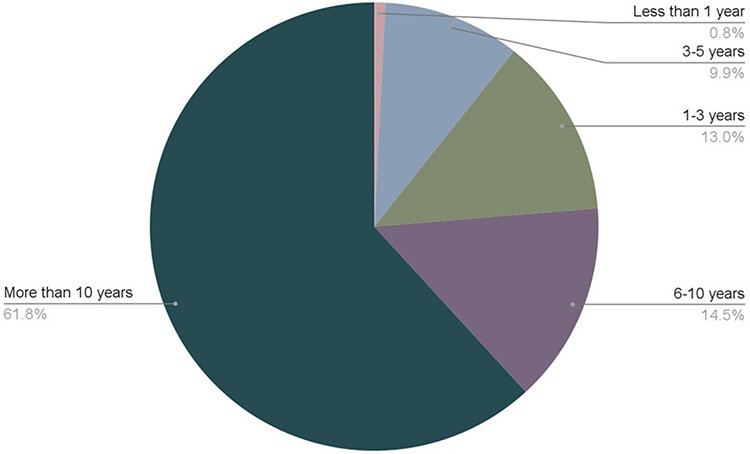
Length of time that ISB community members have been in their career. 131 respondents participated in the survey. The full original dataset is available in Zenodo (2).

**Table 5.** Panelists of the third session of ISB2021 virtual conference; the link to the session recording is available in Table 1

Panelist	Affiliation
Sushma Naithani, Moderator	OSU, Oregon, USA
Laurie Goodman	GigaScience Press
Yasmin Alam-Faruque	Healx
Varsha Khodiyar	Springer Nature

The discussion started with a recap of Picture A Scientist (https://www.pictureascientist.com/), a documentary film that was screened by the ISB EDI in March 2021 (and is currently available on Netflix).

#### Impact of ‘Picture A Scientist’

Picture of Scientist is a documentary that follows three women in different scientific careers: Jane Willenbring, a geologist who faced unrelenting harassment during a once-in-a-lifetime opportunity performing fieldwork in Antarctica; Nancy Hopkins, a biologist who documented concrete evidence of discrimination against women in allocating lab space at her institution, and Raychelle Burks, a chemist who had to contend with a hostile work environment as she progressed through her career. The primary consensus from our panel in response to the movie was a feeling of empathy, commiseration and a recognition that we still need to fight for equity for women in science.

Our panelists called for the need to do more to recognize and acknowledge gender discrimination as well as other forms of unconscious biases that persist in the scientific field. They recognized unique challenges for women, people of color, immigrant scientists, etc. Opportunities to progress in science, particularly in academia, can be very limited without a Ph.D. If someone experiences issues in their lab during their early career training, it can be really difficult to start over. In addition, training can be very specialized and limited. They called out the need for better strategies to aid scientists-in-training and junior scientists when their progress is impeded. These kinds of challenges may not exist in other male-dominated fields like law, where there are opportunities to move between firms.

For those who are dependent on employment visas from immigration offices, they may feel less empowered to take action or speak up when their immigration status is linked to their employment. In addition, the need for recommendations from previous employers may impact our sense of empowerment to take action against inappropriate workplace situations. We all need to stand up and take action when we see discrimination and inappropriate actions. We need to be allies and support each other. However, the problem with implicit bias is that many well-intentioned folks are not aware of their own biases and how it contributes to the environment of scientific institutions, fraternities and societies. Thus, we also need clear institutional guidelines, support for training the scientist in soft skills and addressing the implicit bias for resolving the issues related to EDI.

Our panelists brainstormed some strategies and mechanisms to address some of these problems.

**Education and training**: Regular education and training sessions, such as unconscious bias training are helpful to provide the most up-to-date information. Tests are available that can give insight on your own potential implicit biases.
**Institutions have the responsibility to aid in reporting of harassment and discrimination:** Most institutions over a certain size have a HR department and mechanisms to report harassment or inequity. For example, the company Healx conducts regular surveys to understand employee engagement and satisfaction in the workplace. The survey includes questions around EDI and provides a platform for employees to anonymously report any inequality/harassment issues they may have encountered. When new students and employees are onboarded, they should be informed about processes for reporting issues to HR.
**Mentorship**: Money talks: if women are awarded large grants earlier in their career, this may significantly help their career trajectory and more established biocurators have the opportunity to help train women on how to write good grants. Including women and other scientists, who are marginalized, in formal and informal collaborations and various professional groups will help to achieve inclusion and diversity of the Science, technology, Engineering, and Mathematics (STEM) disciplines.
**Defund offenders**: Institutions and funding agencies should implement policies to take away positions and/or grant funding from people who are guilty of harassment or discrimination.

#### Opportunity for the ISB: define our job titles

The panelists pointed out that standardization of job titles could help with career progression. The ISB has an opportunity to help define standardized job titles across the ranks. For example, what does a starting position look like and what qualifications does a more advanced biocurator typically have? What is the difference between a Lead Biocurator and a Senior Biocurator? Our recent survey revealed that the majority of respondents (62%) have been in their position for 10 years or more, but only about half (49.6%) of the biocurators who responded have been promoted since they started their career in biocuration (Figure 2).

Job titles for biocurators vary widely and there is a lack of standardized names and titles for the biocuration positions. The field of biocuration has existed for approximately 20 years, yet there is not a widespread understanding of what a biocurator does and what a typical career progression should look like.

Based on results from a recent survey that was conducted by the EDI Subcommittee, ISB community members reported 24 unique job titles. Of note, most respondents identified as (bio)curators, but some respondents distinguished their title as a Scientific curator or Scientific Database curator, emphasizing the need for standardization of the job titles.

A generic biocuration position description, available on the ISB website, was created as an outcome of the Careers in Biocuration Workshop at the Biocuration 2018 conference in Shanghai, China. This could be used as a starting point for further definitions and standardization of position descriptions.

#### We need better data

As scientists, we recognize the need for concrete data and evidence to back up assertions and stimulate change. There is a call for the ISB to collect data from the biocuration community to address key questions such as:

- Are women being paid less than men?
- What is the gender breakdown of the membership of the ISB?
- What percentage of women obtain grant funding compared to men?
- Is there evidence of gender or racial discrimination in the biocuration community?
- Are biocurators progressing in their career at the same rate as other types of scientists?

A previously published report on ‘Gender Balance at the International Society for Biocuration Annual Conferences’ is available in Zenodo (3).

### Session 4: strategic planning

The final panel discussion brought together former members of the ISB EC, to recount their goals for the ISB when they served on the committee, their impressions on the current work on the ISB, and any future vision or thoughts on future efforts or strategic goals for the Society. Nicole Vasilevsky, Ph.D., from the University of Colorado and Chair of the Executive Committee of ISB moderated the session and hosted four panelists (outlined in Table 6): Andrew Su, Ph.D., Professor at Scripps Research Institute, California; Mike Cherry, Ph.D., Professor at Stanford University, California; Pascale Gaudet, Ph.D., Senior Project Manager at SIB Swiss Institute of Bioinformatics, Switzerland; and Monica Munoz-Torres, Ph.D., Associate Research Professor at University of Colorado.

**Table 6.** Panelists of the fourth session of ISB2021 virtual conference; the link to the session recording is available in Table 1

Panelist name	Affiliation	Years served on ISB EC
Nicole Vasilevsky, Moderator	University of Colorado Anschutz Medical Campus, Colorado, USA	2017–present (secretary 2017–20, chair 2020–present)
Andrew Su	Scripps Research Institute, California, USA	2016–19
Mike Cherry	Stanford University, California, USA	2010–16 (chair 2015–16)
Pascale Gaudet	SIB Swiss Institute of Bioinformatics, Switzerland	2009–13 (chair 2009–13)
Monica Munoz-Torres	University of Colorado Anschutz Medical Campus, Colorado, USA	2012–17 (secretary 2012–16, chair 2016–17)

When the ISB was formed as a formal professional body, the overarching goal of the Society was to foster the careers and community of biocurators, provide recognition for the work that we do and provide forums and venues for networking and interaction. Our annual international meetings have provided a venue for biocurators to gather and exchange ideas and network. Additionally, the ISB aimed to develop a diverse and inclusive Society. Strides have been made in this direction with our newly established Code of Conduct that was initially created by the EDI committee in 2019 and has been iteratively updated since.

Our Society benefited from the efforts of the ISB EC back in 2008, with the establishment of this journal, Database, that focuses on disseminating publications describing biological databases and curation efforts. Database kindly offers a 20% discount on publication fees to ISB members, who frequently publish their works here. The creation of this specific journal for biocuration was a big achievement for our field—as it was previously challenging to publish in traditional scientific journals—and Database provides our community with a specific venue to publish our research work. The panelists discussed a future opportunity for the ISB to provide microgrants to cover publication costs in Database, in situations where biocurators have limited funding. An additional option would be considering the micropublication system, where no publication cost is involved while still allowing to make research data public (4) (https://www.micropublication.org/).

Finally, there is an opportunity for the ISB to work with publishers to update the instructions to authors and to require them to include official gene nomenclature and outline standards for reagents and accession numbers. Some efforts toward this end have been made by the Resource Identification Initiative, which promotes the use of unique Research Resource Identifiers in the methods sections of research publications (5).

One important aspect of the ISB that may be less visible to the community is the IT infrastructure. Andrew Su initially set up a lot of the technology that is being used by the EC now, including our website and our membership tracking and payment system. There are ongoing needs for website support and technological expertise in the EC, and we encourage anyone with these skills to consider running for election to the EC in the future or volunteering with the Society.

One of the recent achievements with the current EC is our subcommittee structure. The ISB has several subcommittees which are described on our website, and this allows for distribution of efforts among the EC members and gives people an opportunity to get engaged outside of the EC. External community members are always encouraged to get involved.

The ISB currently offers two awards for Exceptional Contribution to Biocuration Award and Biocuration Award. We recently created an additional award for Biocuration Inclusivity that is designed to assist individual ISB members in attending events which they would not otherwise be able to be present at for reasons beyond simple lack of funding for registration, travel and accommodation. It was proposed to either add additional awards or to assign them more freqeuntly, in order to provide more recognition to our community members and push out visibility for our Society. They may not be getting recognition in other ways like other academics through publications and promotions.

Panelists commended the recent efforts to support and promote career development for biocurators, with a great starting point being the establishment of formal training opportunities and professional certificates that did not previously exist in the field. At the same time, creating, maintaining and sharing FAIR training materials—GOBLET (https://www.mygoblet.org/) and ELIXIR TeSS (https://tess.elixir-europe.org/)—should be even more supported and pursued, while also providing dedicated learning sessions where to present them. One future goal of the ISB is to create a system to encourage development of Open Educational Resources that focus on specific topics, such as licensing or Gene Ontology annotations, and make these resources openly and freely available to the community.

All these directions will play a crucial role in our job security and will make room for professional development of biocuration careers, actively supported by the ISB.

## Future meetings

This 2021 virtual conference received great feedback overall, as it has been more accessible for attendees, particularly for those who could not afford to travel e.g. due to family commitments. It was therefore proposed to keep on maintaining some virtual events even once the restrictions related to the pandemic will be lifted and the conference will resume in-presence. One suggestion was to hold monthly webinars with rotating speakers each month. The EC will consider an invited speaker monthly webinar series in the future, with solicited recommendations for speakers from the community, as an additional way to provide opportunities for promotion and recognition of our work. In 2022, the main conference (ISB2022) will be held virtually, featuring three virtual sessions with talks and panel discussions starting April 2022. A small, local meeting will be held in the UK in May 2022 for biocurators in the region.
